# Le nodule de Sœur Marie Joseph, une rare métastase contributive

**DOI:** 10.11604/pamj.2015.22.130.5499

**Published:** 2015-10-13

**Authors:** Mohammed Ouazni, Souhail Ahmimech

**Affiliations:** 1 Clinique Chirurgicale J, Institut National d'Oncologie Rabat, UPR de Chirurgie Générale, Faculté de Médecine et de Pharmacie, Université Mohammed V Souissi, Rabat, Maroc

**Keywords:** Hernie ombilicale, kyste de l′ouraque, déplissement ascitique de l′ombilic, umbilical hernia, Urachal cyst, Ascitic unfolding of the umbilicus

## Image en medicine

Les métastases cutanées ombilicales sont rares et habituellement associées à un adénocarcinome intra-abdominal en particulier les carcinomes gastriques. Nous rapportons l'observation d'une patiente de 80 ans admise pour une masse abdominopelvienne compliquée d'une ascite de grande abondance d'origine carcinomateuse à la cytologie évoluant dans un contexte d'altération de l'état général. La TDM abdominale a conclue à une ascite d'origine carcinomateuse avec des stigmates de carcinome péritonéale généralisée dont l'étiologie ne pouvait être déterminée. L'examen clinique entrepris lors de l'hospitalisation trouve une masse de l'ombilic polylobé d'aspect bleuâtre, poreuse au niveau de la fossette ombilicale et déformant complètement l'ombilic, saignante au toucher. Un prélèvement biopsique sur cette masse a été effectué et l'étude histologique était en faveur d'un adénocarcinome peu différencié de l'ovaire. Il s'agissait en effet d'un nodule de S'ur Marie Joseph. Une découverte clinique qui est d'une aide inestimable orientant le diagnostic étiologique et la prise en charge thérapeutique et épargnant à la patiente une intervention chirurgicale d'exploration sur un terrain fragilisé par sa pathologie maligne. Nous attirons l'attention des cliniciens: oncologue et chirurgiens à cette entité clinique, qui est rare certes, mais très contributive au diagnostic étiologique et par conséquent à une prise en charge optimale pour ce genre de patient.

**Figure 1:**
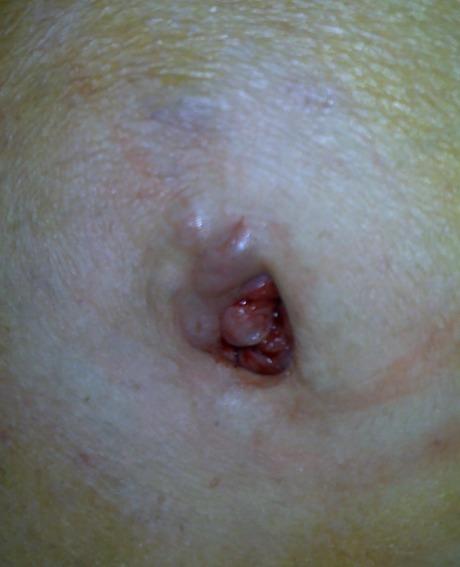
Fossette ombilicale comblée par la greffe métastatique ovarienne saignant facilement à la manipulation

